# Efficacy of front‐line immunochemotherapy for transplant‐ineligible mantle cell lymphoma: A network meta‐analysis of randomized controlled trials

**DOI:** 10.1002/cam4.6183

**Published:** 2023-06-01

**Authors:** Caixia Jing, Ailin Zhao, Jinjin Wang, Ting Niu

**Affiliations:** ^1^ Department of Hematology, West China Hospital Sichuan University Chengdu China

**Keywords:** immunochemotherapy, mantle cell lymphoma, network meta‐analysis, OS, PFS

## Abstract

**Background:**

There is no standard first‐line immunochemotherapy regimen for transplant‐ineligible patients with mantle cell lymphoma (MCL) currently, and the efficacy of various treatment remains unclear.

**Methods:**

We conducted a Bayesian network meta‐analysis (NMA) of all eligible randomized controlled trials. Pairwise comparisons and ranking of different first‐line treatment options were performed.

**Results:**

Nine studies were included in the NMA, involving a total of 2897 MCL patients. The BR‐Ibrutinib+R regimen showed the best progression‐free survival (PFS), with a surface under the cumulative ranking curve (SUCRA) of 0.89 and probability of being the best treatment (PbBT) of 69%. The VR‐CAP regimen was the most potential intervention to improve overall survival (OS), with a SUCRA of 0.89 and PbBT of 63%. Compared with the R‐CHOP regimen, the BR regimen achieved a better PFS (hazard ratio [HR] 0.45 [95% credible interval 0.2–0.96]). The BR‐Ibrutinib+R regimen (HR 0.14 [0.02–0.99]), BR+R regimen (HR 0.19 [0.034–0.99]), and BR regimen (HR 0.3 [0.08–1.03]) were superior to CHOP regimen with better PFS. The R‐FC regimen (HR 2.27 [1.01–5.21]) or FC regimen (HR 3.17 [1.15–8.71]) was inferior to the VR‐CAP regimen with a worse OS.

**Conclusions:**

Our study presents the most promising first‐line treatment strategy for transplant‐ineligible MCL patients in terms of PFS and OS, which provides innovative treatment strategy for MCL treatment.

## 1 INTRODUCTION

Mantle cell lymphoma (MCL) is a rare non‐Hodgkin's lymphoma, accounting for approximately 5%–7% of lymphomas. It is a late‐onset disease with a median age at diagnosis of 60–70 years and mainly occurs in males (male: female ratio approximately 3:1).^1^ In addition, MCL is highly heterogeneous, with different pathophysiological and genetic features, clinical manifestations, staging, and prognosis. Some patients can undergo a wait‐and‐see observative management without immediate treatment on diagnosis, while others are highly aggressive, and the efficacy of current first‐line treatment is far from satisfactory. Initial therapy for MCL patients can be classified as transplant‐eligible or transplant‐ineligible, depending on the age, and severity of the comorbidities of the patients. Because the majority of patients are of advanced age, the opportunity for transplantation is limited.^2^

In the past 20 years, there have been significant advances in the prognosis of MCL patients because of the introduction of rituximab. However, there is still no cure, and the relapse is inevitable. The earlier MCL patients progress after the first‐line therapy, the worse the prognosis, especially for those who progress within 6 months after first‐line therapy.^3^ Patients need better first‐line treatment to improve the clinical outcome, especially high‐risk patients. Currently, there are a number of first‐line treatments available for patients with MCL, but it remains unclear which one may work best.^4^ Clinical drug selection is based on the availability of drugs, patients' conditions, doctors' clinical experience, and other factors. As new targeted therapeutic agents enter first‐line therapy, such as ibrutinib and bortezomib,^5^, ^6^ it remains unclear whether they will further improve patient outcomes. A comprehensive comparison of these schemes is necessary.

In practice, it is difficult to directly compare multiple treatment options, but indirect comparison is also a strategy that may provide useful information for clinical treatment choices. Network meta‐analysis (NMA) can compare the relative treatment effects of multiple interventions by synthesizing evidence from a network of randomized controlled trials (RCTs), which can be very useful for the choice of clinical treatment plans. The studies included in NMA must conform to similarity and transitivity. Indirect comparison results are valid only when the quality and other characteristics of the studies are similar enough.^7^ Therefore, only RCTs were included in our study. There are few studies on patients with transplant‐eligible MCL that can be included in the NMA,^8^, ^9^, ^10^, ^11^ and clinical practicability is insufficient. Thus, we only performed a NMA in transplant‐ineligible MCL to compare the efficacy of first‐line immunochemotherapy regimens to help clinical selection of the best treatment.

## 2 METHODS

### 2.1 Search methods and selection criteria

This article was conducted in accordance with the Preferred Reporting Items for Systematic Reviews and Meta‐Analyses (PRISMA) 2020 standards^12^ and registered with the PROSPERO International Prospective Register of Systematic Reviews (CRD42022385098). A systematic search was conducted in November 20, 2022 to December 31, 2022, including PubMed, Embase, Web of Science, Cochrane Database of Systematic Reviews, Cochrane Central Register of Controlled Trials, and ClinicalTrials.gov. The main keywords searched were as follows: (“Lymphoma, Mantle‐Cell” OR “mantle cell lymphoma”) AND (“Therapeutics” OR “therapy”) AND (“randomized controlled trial” OR “randomized). Included articles must meet all of the following criteria: (1) Patients with untreated/newly MCL diagnosed by histopathologic biopsy. (2) First‐line treatment options were compared. (3) The study was a randomized controlled trial. (4) Progression‐free survival (PFS) or overall survival (OS) can be obtained. (5) Published in English. For overlapping studies and updates, we selected the latest data or studies with the largest sample size.

### 2.2 Data extraction

The following information was extracted from all included studies: authors, publication year, title, country, registration number, stage, age, gender, treatment arms, patient number, and hazard ratio (HR) and 95% confidence interval (CI) for PFS and OS. If HR was not reported, it was calculated using the calculations spreadsheet provided by Tierney et al.^13^ After eliminating duplicate studies, two researchers (CJ and AZ) independently selected the articles and extracted the article data by reading the titles, abstracts and full texts. If the two disagreed, a consensus was reached with the third author (JW).

### 2.3 Risk of bias assessment

Two reviewing authors (CJ and AZ) independently used the Version 2 of the Cochrane tool for assessing risk of bias in randomized trials (RoB 2) to evaluate the quality of the included studies.^14^ The two reviewing authors' conclusions must be identical, or the third review author (JW) resolved their inconsistencies.

### 2.4 Network meta‐analysis

A Bayesian hierarchical model was used for NMA in this study. The primary endpoint was PFS, defined as the time from random assignment or induction therapy start to disease progression, relapse, or death from any cause. The secondary endpoint was OS, defined as the time from random assignment or induction therapy start to death from any cause.

There was no way to compare the consistency assumption using node‐splitting analysis, because the network did not have a closed loop. The random effect model was used in the NMA, and heterogeneity was evaluated by I^2^. Posterior samples were generated using the Markov Chain Monte Carlo methods running in three chains. We set up 80,000 adaptation iterations and 10,000 simulation iterations, and checked convergence by trace plots. The league tables rendered the pairwise comparisons of HRs of two different treatments. In addition, we ranked interventions by calculating the surface under the cumulative ranking curve (SUCRA). SUCRA values ranged from 0 to 1, with the closer to 1, the more likely to be the most effective intervention. We also calculated the probability of being the best treatment (PbBT) for each candidate treatment.^15^ To assess the robustness of the statistical inference, sensitivity analyses were performed using the following methods, for PFS: (i), excluding the data from Fischer L et al. on the post hoc assessment of the treatment effect between R‐CHOP (rituximab, cyclophosphamide, doxorubicin, vincristine, and prednisone) regimen and CHOP (cyclophosphamide, doxorubicin, vincristine, and prednisone) regimen; (ii) from (i), further excluding the data from Flinn IW. et al. with small sample size; (iii) from (ii), further excluding the data from Smith MR. et al., because its HR and 95% CI were calculated by the methods provided in the literature; for OS: (i) as with PFS, excluding the data from Fischer L et al.; (ii) from (i), further excluding the data from Rule S et al. on the treatment effect estimation between R‐FC (rituximab, fludarabine, and cyclophosphamide) regimen and FC (fludarabine and cyclophosphamide) regimen; (iii) from (ii), further excluding the data from Kluin‐Nelemans HC. et al. on the treatment effect estimation between R‐FC regimen and R‐CHOP regimen. All data analyses were performed using R (version 4.2.2) and JAGS (version 4.3.1) software.

## 3 RESULTS

### 3.1 Study selection

**FIGURE 1:**
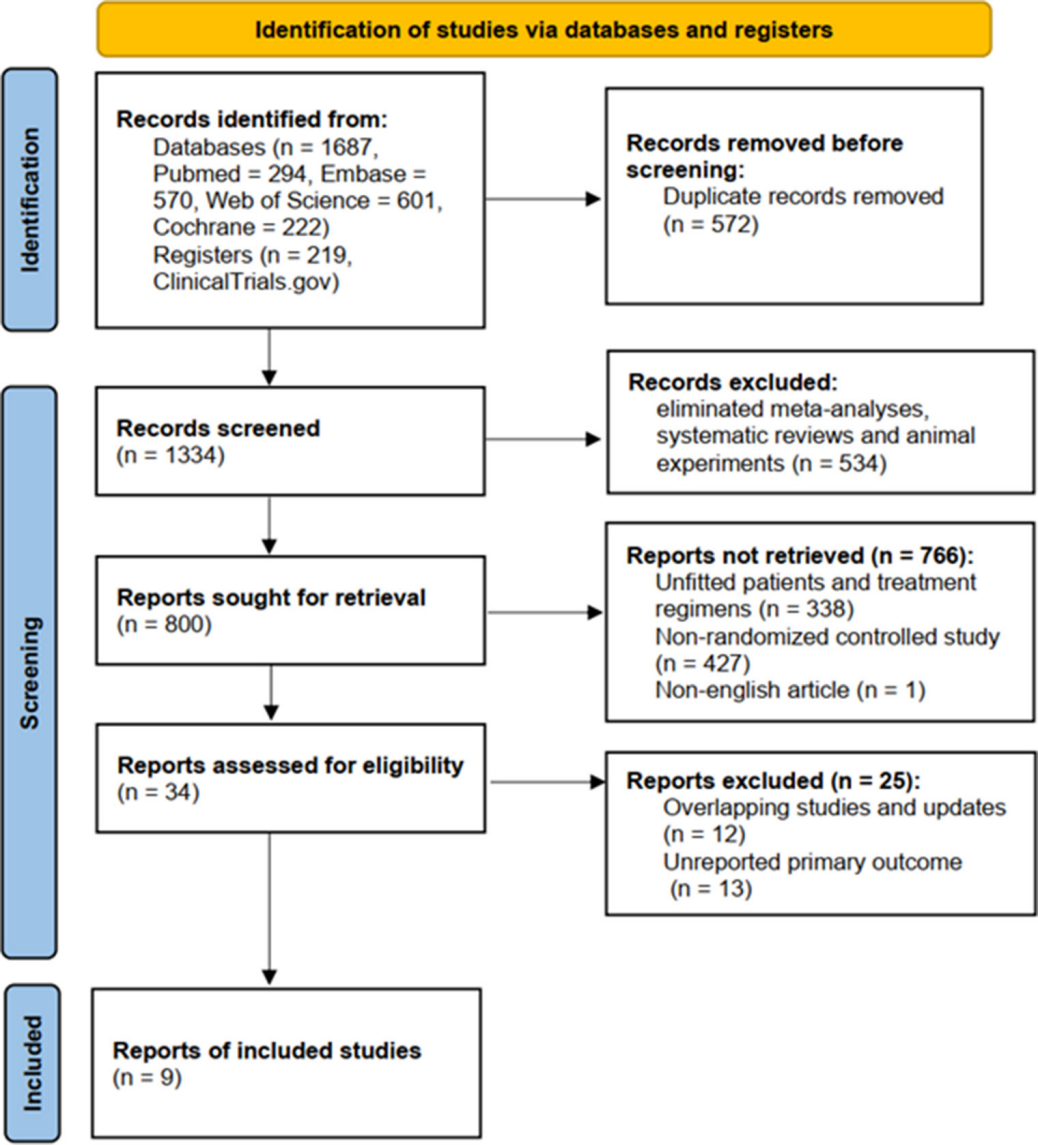
PRISMA flowchart of the identified and included studies.

The flowchart of literature screening is shown in Figure 1. A total of 1906 initial studies were retrieved. After removing duplicate studies, 1334 studies remained. We then excluded 534 meta‐analyses, systematic reviews, and animal studies. Another 766 publications were eliminated by reading abstracts and titles. Because we focused on the overall primary therapy, we excluded comparative data on maintenance therapy where the induction regimen was unknown. Finally, nine studies were included in the NMA, involving a total of 2897 MCL patients. Six studies were full texts,^5^, ^6^, ^16^, ^17^, ^18^, ^19^, ^20^ and three studies were conference abstracts.^21^, ^22^, ^23^

### 3.2 Study characteristics

**FIGURE 2:**
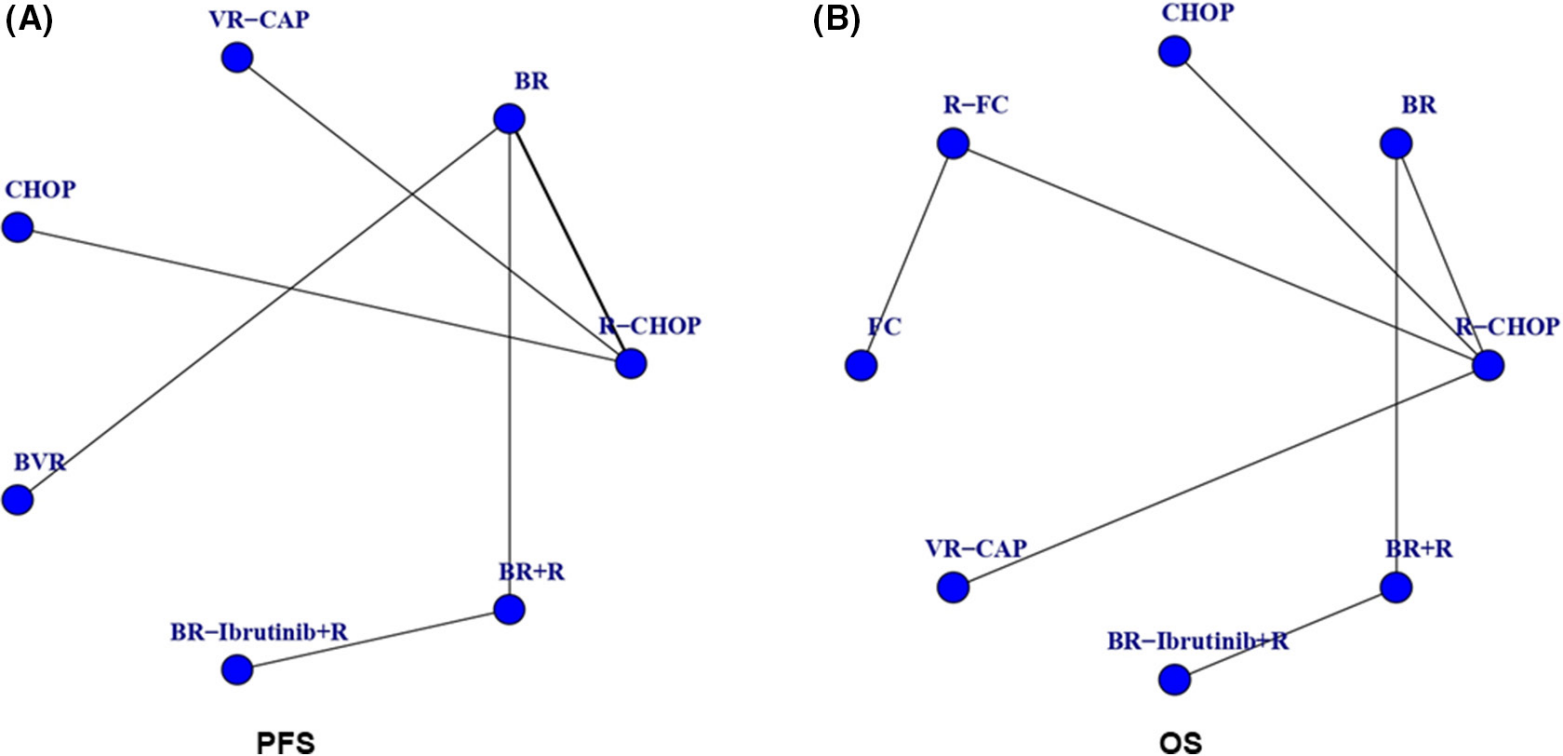
Network meta‐analysis of eligible comparisons. The network interventions regarding (A) PFS (progression‐free survival), and (B) OS (overall survival). The more studies of each intervention, the bigger the dots, and the more direct comparisons, the thicker the lines. R‐FC: rituximab, fludarabine, and cyclophosphamide; R‐CHOP: rituximab, cyclophosphamide, doxorubicin, vincristine, and prednisone; CHOP: cyclophosphamide, doxorubicin, vincristine, and prednisone; FC: fludarabine and cyclophosphamide; VR‐CAP: bortezomib, rituximab, cyclophosphamide, doxorubicin, and prednisone; BR: bendamustine and rituximab; BVR: bendamustine, bortezomib, and rituximab; BR‐Ibrutinib+R: bendamustine, rituximab, ibrutinib, and rituximab maintenance; BR + R: bendamustine, rituximab, and rituximab maintenance.

The characteristics and efficacy data of the included RCTs are summarized in Table 1. All trials were published between 2012 and 2022. The median age at diagnosis of MCL patients in the included studies ranged from 61 to 71 years. The median follow‐up time varied from 3.08 to 13.4 years. The results of the risk of bias assessment can be found in Figure S1. One study was assessed as low risk, and the other eight studies were rated as some concerns. Figure 2 shows the graph of the evidence network for PFS and OS. The comparisons of PFS involved seven different immunochemotherapy and maintenance regimens, including R‐CHOP, BR (bendamustine and rituximab), VR‐CAP (bortezomib, rituximab, cyclophosphamide, doxorubicin, and prednisone), CHOP, BVR (bendamustine, bortezomib, and rituximab), BR‐Ibrutinib+R (bendamustine, rituximab, ibrutinib, and rituximab maintenance), and BR + R (bendamustine, rituximab, and rituximab maintenance). For OS comparisons, there were eight different treatment regimens, including R‐CHOP, BR, VR‐CAP, CHOP, R‐FC, FC, BR‐Ibrutinib+R, and BR + R.

**TABLE 1:** Characteristics of the studies included.

Data source	Stage	Median age (range)	Male	Median follow‐up	Primary endpoint	Arms	Patient number	PFS HR (95% CI)	OS HR (95% CI)
Kluin‐Nelemans et al. 2012^17^	II–IV	70 years (60–87)	70%	3.08 years	CR	R‐FC^a^	246	NA	1.5 (1.13–1.99) *p* = 0.005
						R‐CHOP^a^	239		
Fischer et al. 2021^23^	Advanced stage	61 years (37–86) vs. 62 years (35–84)	72% vs. 79%	13.4 years	FFS and OS	R‐CHOP	184	0.67 (0.53–0.86)^b^ *p* = 0.0012	0.78 (0.61–0.99)^b^ *p* = 0.039
						CHOP	201		
Rule et al. 2016^18^	I–IV	66 years (37–85) vs. 66 years (36–88)	79.3% vs 73.7%	6.02 years	OS	R‐FC	186	0.54 (0.42–0.69) *p* < 0.001	0.72 (0.55–0.94) *p* = 0.016
						FC	184		
Robak et al. 2015, 2018^6^, ^16^	II–IV	65 years (26–88) vs. 66 years (34–82)	73% vs. 75%	3.33 years	PFS	VR‐CAP	243	0.63(0.5–0.79) *p* < 0.001	0.66 (0.51–0.85)^c^ *p* = 0.001
						R‐CHOP	244		
Rummel et al. 2013^19^	II–IV	70 years (64·5–74)	NA	3.75 years	PFS	BR	46	0.49 (0.28–0.79) *p* = 0.0044	NA
						R‐CHOP	48		
Smith et al. 2021^21^	NA	67 years (42–90)	73%	4.25 years	PFS	BVR^d^	179	2‐year PFS 79.6% (95% CI 73.8–85.9) vs. 74.5% (95% CI 68.2–81.4) *p* = 0.268	NA
						BR^d^	180		
Flinn et al. 2019^20^	II–IV	66 years vs. 68 years	89% vs. 78%	5.42 vs. 5.34 years	CR	BR	37	0.40 (0.21–0.75) *p* = 0.0035	0.86 (0.40–1.83) *p* = 0.6894
						R‐CHOP^e^	37		
Wang et al. 2022^5^	II–IV	71 years (65–86) vs. 71 years (65–87)	68% vs. 71%	7.06 years	PFS	BR‐Ibrutinib+R	261	0.75 (0.59–0.96) *p* = 0.01	1.07 (0.81–1.40)
						BR + R	262		
Rummel et al. 2016^22^	II–IV	70 years	NA	4.52 years	PFS	BR + R	59	0.64 (0.36–1.14) *p* = 0.13	1.53 (0.73–3.32) *p* = 0.271
						BR	61		

Abbreviations: BR + R, bendamustine, rituximab, and rituximab maintenance; BR, bendamustine and rituximab; BR‐Ibrutinib+R, bendamustine, rituximab, ibrutinib, and rituximab maintenance; BVR, bendamustine, bortezomib, and rituximab; CHOP, cyclophosphamide, doxorubicin, vincristine, and prednisone; CI, confidence interval; CR, complete response; FC, fludarabine and cyclophosphamide; FFS, failure‐free survival; HR, hazard ratio; NA, not available; OS, overall survival; PFS, progression‐free survival; R‐CHOP, rituximab, cyclophosphamide, doxorubicin, vincristine, and prednisone; R‐FC, rituximab, fludarabine, and cyclophosphamide; VR‐CAP, bortezomib, rituximab, cyclophosphamide, doxorubicin, and prednisone.

^a^ Patients who responded to induction regimen were randomized to rituximab or interferon maintenance therapy.

^b^ Data adjusted by Mantle Cell Lymphoma International Prognostic Index (MIPI).

^c^ The median follow‐up time was 6.83 years.

^d^ Patients who responded to induction regimen were randomized to rituximab or lenalidomide plus rituximab consolidation therapy.

^e^ Some patients received R‐CVP regimen.

### 3.3 Ranking for regimens based on PFS and OS

**FIGURE 3:**
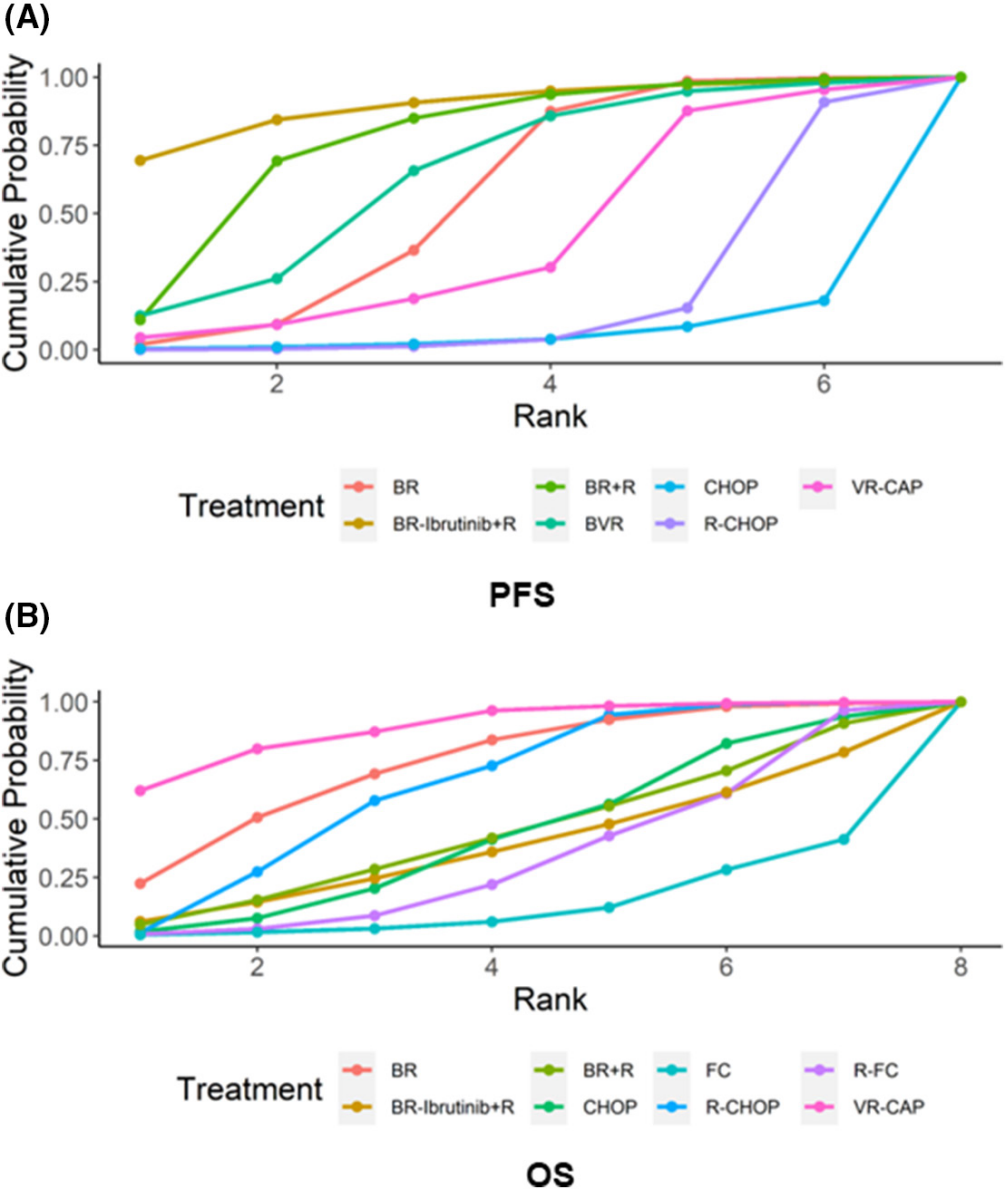
Surface under the cumulative ranking (SUCRA) curves. SUCRA curves regarding (A) PFS and (B) OS. SUCRA values range from 0 to 1, with the closer to 1, the more likely to be the most effective intervention.

SUCRA and PbBT for each treatment strategy are shown in Tables 2 (PFS) and 3 (OS). The SUCRA curves for PFS and OS are shown in Figure 3. BR‐Ibrutinib+R regimen was recognized as the therapy that resulted in the best PFS, with a SUCRA of 0.89 and PbBT of 69%, followed by BR + R regimen (SUCRA 0.76, PbBT 11%) and BVR regimen (SUCRA 0.64, PbBT 13%). VR‐CAP regimen was the most likely intervention to improve OS in MCL patients, with a SUCRA of 0.89 and PbBT of 63%, followed by BR regimen (SUCRA 0.74, PbBT 22%) and R‐CHOP regimen (SUCRA 0.65, PbBT 1%).

**TABLE 2:** Ranking of progression‐free survival of immunochemotherapy regimens.

Regimen	SUCRA	PbBT
BR‐Ibrutinib + R	0.89	69%
BR + R	0.76	11%
BVR	0.64	13%
BR	0.56	2%
VR‐CAP	0.41	4%
R‐CHOP	0.19	0%
CHOP	0.05	0%

Abbreviations: SUCRA: surface under the cumulative ranking curve; PbBT: probability of being the best treatment.

**TABLE 3:** Ranking of overall survival of immunochemotherapy regimens.

Regimen	SUCRA	PbBT
VR‐CAP	0.89	63%
BR	0.74	22%
R‐CHOP	0.65	1%
BR + R	0.44	5%
CHOP	0.43	2%
BR‐Ibrutinib + R	0.38	6%
R‐FC	0.33	1%
FC	0.13	0%

Abbreviations: PbBT, probability of being the best treatment; SUCRA, surface under the cumulative ranking curve.

### 3.4 Pairwise comparison based on PFS and OS

**FIGURE 4:**
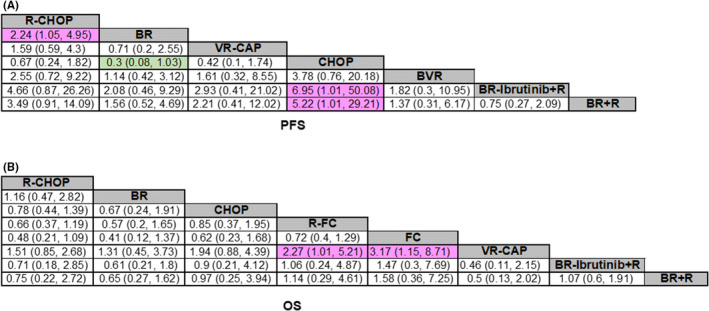
League tables of network meta‐analysis results. The images show the pairwise comparison of different treatments based on (A) PFS and (B) OS. Hazard ratio (HR) values are rendered at the intersection of rows and columns. A HR of >1 favors row‐defining treatment.

**FIGURE 5:**
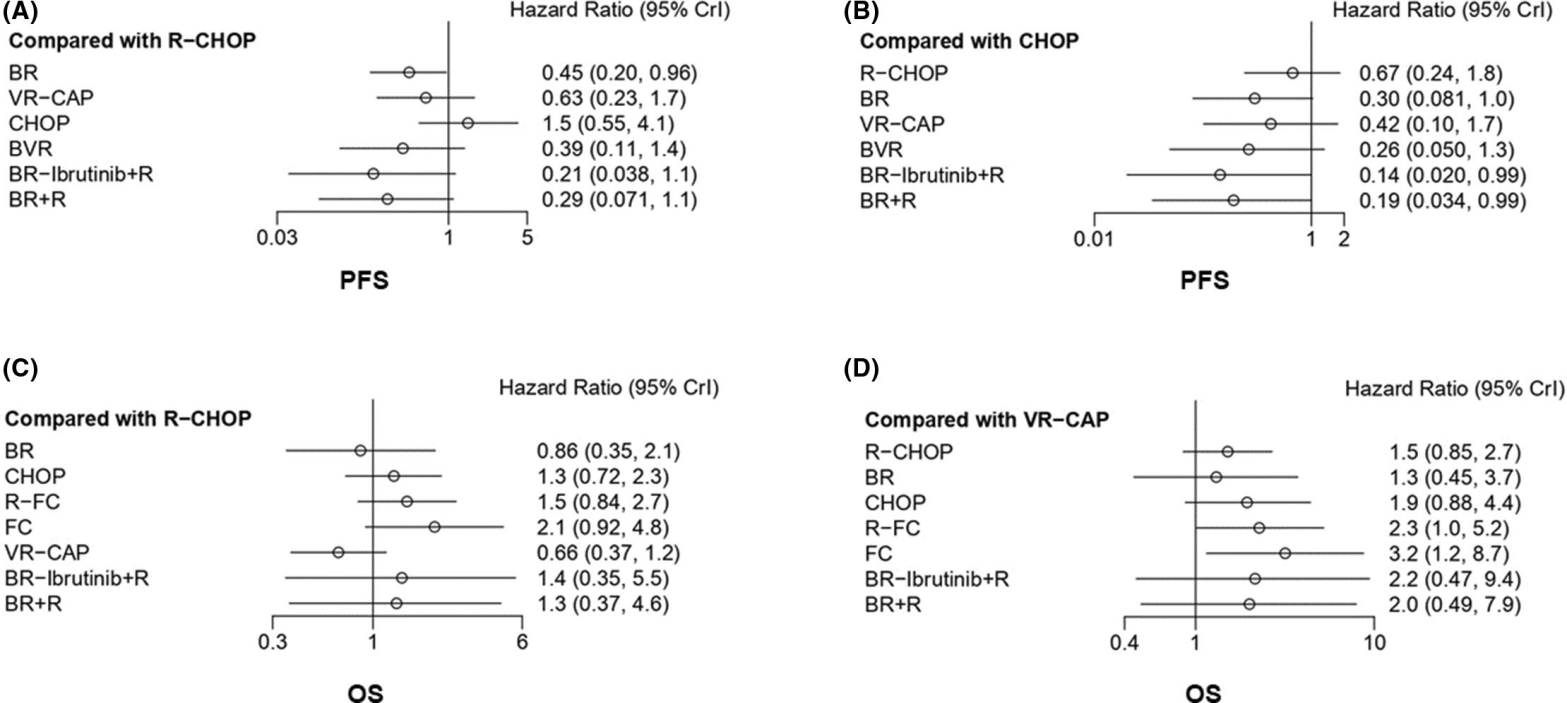
Forest plots of network meta‐analysis of all studies. (A) Forest plot of hazard ratios (HRs) for PFS of other treatments compared with R‐CHOP regimen; (B) Forest plot of HRs for PFS of other treatments compared with CHOP regimen; (C) Forest plot of HRs for OS of other treatments compared with R‐CHOP regimen; (D) Forest plot of HRs for OS of other treatments compared with VR‐CAP regimen. CrI, credible interval.

All results of PFS or OS pairwise comparison among different treatments are shown in league tables (Figure 4). In order to more intuitively show the results of pairwise comparison with significant differences, we mapped forest plots with specific treatment regimens as controls (Figure 5). For PFS, comparisons of other regimens with R‐CHOP or CHOP regimen are shown in Figure 5A,B. Compared with R‐CHOP regimen, BR regimen achieved significantly better PFS (HR 0.45 [95% credible interval (CrI) 0.2–0.96]). BR‐Ibrutinib+R regimen (HR 0.14 [0.02–0.99]), BR + R regimen (HR 0.19 [0.034–0.99]), and BR regimen (HR 0.3 [0.08–1.03]) were superior to CHOP regimen. For OS, comparisons of other regimens with R‐CHOP, or VR‐CAP regimen are presented in Figure 5C,D. None of the other regimens showed significant differences in OS compared to R‐CHOP regimen, whereas VR‐CAP regimen may achieve better OS. R‐FC regimen (HR 2.27 [1.01–5.21]) or FC regimen (HR 3.17 [1.15–8.71]) was a worse therapy with inferior OS compared with VR‐CAP regimen. For heterogeneity, I^2^ was 8% for PFS and 14% for OS, indicating that the included studies had good homogeneity.

### 3.5 Sensitivity analysis

We performed sensitivity analyses as described in the methodology section. The results are summarized in Tables S1,S2 and Figures S2–S4. BR‐Ibrutinib+R regimen remained the best strategy regarding PFS, followed by BR + R, BVR, BR, VR‐CAP, and R‐CHOP regimen. For OS, VR‐CAP regimen also remained the best immunochemotherapy regimen, followed by BR, R‐CHOP, BR + R, BR‐Ibrutinib+R, R‐FC, and FC regimen. All the results were similar, proving that the reported results were robust.

## 4 DISCUSSION

At present, the National Comprehensive Cancer Network (NCCN) and the European Society for Medical Oncology (ESMO) guidelines both recommend multiple immunochemotherapy regimens, such as R‐CHOP, BR, R‐BAC, and VR‐CAP, as the first‐line treatments for patients with transplant‐ineligible MCL.^1^, ^24^ An increasing number of new targeted agents are showing good efficacy in relapsed/refractory MCL.^25^ Therefore, many researchers have tried to introduce these targeted therapy drugs into first‐line therapy, hoping to bring better curative effects to MCL patients.^26^, ^27^, ^28^ In this context, the choice of treatments for untreated/newly MCL patients becomes more complex and difficult. It is almost impossible to make a head‐to‐head comparison between two treatments that include the latest drugs. In this case, NMA is a useful and critical statistical method to indirectly compare multiple treatment regiments and rank them.

Our study is the first NMA of first‐line treatment options for transplant‐ineligible MCL patients. The reason why there are few studies in this field may be the lack of data and relevant modern regimens, which leads to difficulty in network construction. The increasing number of studies related to first‐line treatments of MCL in recent years has enabled us to further analyze these data. Although our study was less heterogeneous, we chose a random effects model to provide more conservative results. Sensitivity analyses also confirmed that our results were stable and reliable. Ranking results can serve as important reference data for clinicians to choose the best treatment for MCL patients. Of course, clinical decision‐making is complex and comprehensive and needs to consider many factors, such as side effects, complications, patient opinions, quality of life, and costs. Efficacy is not the only determinant.

Our results showed that BR‐Ibrutinib+R regimen (SUCRA 0.89, PbBT 69%) was most likely to bring longer PFS to MCL patients. This treatment regimen was based on the BR + R regimen with the addition of ibrutinib. Adding ibrutinib extended the median PFS of untreated/newly MCL patients for whom transplantation was not feasible by 2.3 years (6.7 years vs. 4.4 years).^5^ Ibrutinib, an inhibitor of Bruton's tyrosine kinase, has been approved for the treatment of chronic lymphocytic leukemia, MCL, Waldenström's macroglobulinemia, etc. It is a promising new antitumor drug. In the treatment of patients with relapsed/refractory MCL, both single drugs, and combinations have shown surprising efficacy.^29^, ^30^, ^31^ Our results provide more evidence for the use of ibrutinib in initial therapy for MCL patients. Ibrutinib is reasonable and attractive for use in very high risk MCL patients, such as those with *TP53* mutation or deletion and blastoid and pleomorphic cytologic patterns. Of course, more clinical trials are needed to confirm this finding. However, it should be noted that the addition of ibrutinib did not prolong the OS of MCL patients. The possible reason is that the addition of ibrutinib does bring more adverse reactions, such as bleeding complications, atrial fibrillation, and infections.^32^, ^33^ In the current era of the COVID‐19 pandemic, infections may have a more adverse impact on patients' OS. In addition, the number of MCL patients receiving second‐line treatment in the ibrutinib group was not the same as that in the control group, and the treatment modalities were not comparable in Wang ML. et al.'s study. Drug resistance in the treatment of MCL with ibrutinib may also be a factor.^34^, ^35^

Our results suggested that VR‐CAP regimen (SUCRA 0.89, PbBT 63%) may be the most effective treatment regimen for prolonged OS in MCL patients. This treatment strategy was to replace vincristine with bortezomib on the basis of R‐CHOP regimen. Bortezomib, a proteasome inhibitor, has shown promising efficacy as a single agent in relapsed/refractory MCL and was approved by the U.S. Food and Drug Administration (FDA) for the treatment of relapsed MCL patients in 2006.^36^, ^37^ Nevertheless, in our study, bortezomib did not appear to have an advantage in prolonging PFS in MCL patients. It may be influenced by the small number of studies included in the network. In addition, one thing to note is that the results of OS may be biased due to the influence of different follow‐up times, unbalanced salvage therapy, and other factors. Hence, the results of PFS may be more important.

Currently, R‐CHOP and BR regimen are the two most commonly used first‐line therapies for MCL patients who are not suitable for transplantation. In the United States, there is a trend from R‐CHOP to BR regimen in the treatment of this subset of patients.^11^ Our results also suggested that BR regimen may provide better survival for MCL patients than R‐CHOP regimen. A previous meta‐analysis showed that R maintenance could improve PFS and OS in MCL patients,^38^ and guidelines also recommend R maintenance therapy for MCL patients in remission after first‐line therapy. However, whether R maintenance after BR regimen is beneficial is still controversial. A prospective randomized controlled trial found that R maintenance after BR regimen did not improve PFS and OS in patients with MCL.^22^ Recently, two real‐world studies of large samples found that R maintenance after BR regimen had a longer time to the next treatment and OS than the observation group.^39^, ^40^ Our results showed that BR+R regimen may have better PFS than BR regimen, but OS did not improve. A growing body of observational data supports R maintenance after BR therapy in elderly patients with MCL who do not receive autologous stem cell transplantation.

Our study overcame the disadvantages of other RCTs which could only compare two regimens at the same time and could not compare multiple regimens involving new drugs. In addition, our study is the first to identify the likely best regimen for PFS or OS for transplant‐ineligible MCL patients in a variety of regimens containing new drugs. Of course, there were some limitations in our study. First, we only included nine studies in our NMA, due to the limited number of original publications of related RCTs. This made our consistency test difficult to implement. This may also be responsible for the fact that most of the pairwise comparisons did not show statistically significant differences. Second, whether MCL patients should receive maintenance therapy after first‐line treatment and what regimen is used for maintenance are questions of great concern to hematologists. With the introduction of innovative drugs such as lenalidomide, bortezomib, and obinutuzumab into maintenance therapy, patient outcomes may be further improved.^26^, ^41^, ^42^ It was unfortunate that our study was unable to analyze more maintenance treatments due to the limited number of the eligible published RCTs. Finally, our study only focused on efficacy, without considering adverse events, cost‐effectiveness, and other important issues.

In conclusion, our findings suggest that BR‐Ibrutinib+R regimen could be the first choice for improving the PFS of MCL patients who are not candidates for transplantation in first‐line treatment. VR‐CAP regimen may be better than other options in improving OS in MCL patients. These results suggest that a regimen containing novel antitumor agents improves outcomes in patients with MCL. There are also many new antitumor drugs in the pipeline that hope to shed new light on the survival of MCL patients. We will continue to monitor these findings to refine our data. In a situation where first‐line immunochemotherapy regimens are difficult to select in MCL patients who are not suitable for transplantation, we hope that our results will help inform clinical treatment decisions.

## AUTHOR CONTRIBUTIONS

**Caixia Jing:** Data curation (equal); formal analysis (equal); investigation (equal); methodology (equal); writing – original draft (equal). **Ailin Zhao:** Data curation (equal); formal analysis (equal); investigation (equal); writing – review and editing (equal). **Jinjin Wang:** Formal analysis (equal); investigation (equal); methodology (equal); writing – original draft (equal). **Ting Niu:** Conceptualization (equal); funding acquisition (equal); supervision (equal); writing – review and editing (equal).

## CONFLICT OF INTEREST STATEMENT

The authors report no conflict of interest.

## ETHICS STATEMENT

This is a meta‐analysis. The Ethical Committee of Sichuan University has confirmed that no ethical approval is required.

## Supporting information


Figure S1.
Figure S2.
Figure S3.
Figure S4.

Table S1‐2.

## Data Availability

The data that support the findings of this study are available from the corresponding author upon reasonable request.
