# Supplementary material for: Efficacy of front‐line immunochemotherapy for transplant‐ineligible mantle cell lymphoma: A network meta‐analysis of randomized controlled trials
Source: Cancer Med. 2023 Jun 1;12(14):15107–16. doi: 10.1002/cam4.6183 (PMC10417079; doi:10.1002/cam4.6183)
Supplement: Supplementary file 1 — Figure S1. Figure S2. Figure S3. Figure S4. [file CAM4-12-15107-s001.pdf]

a)

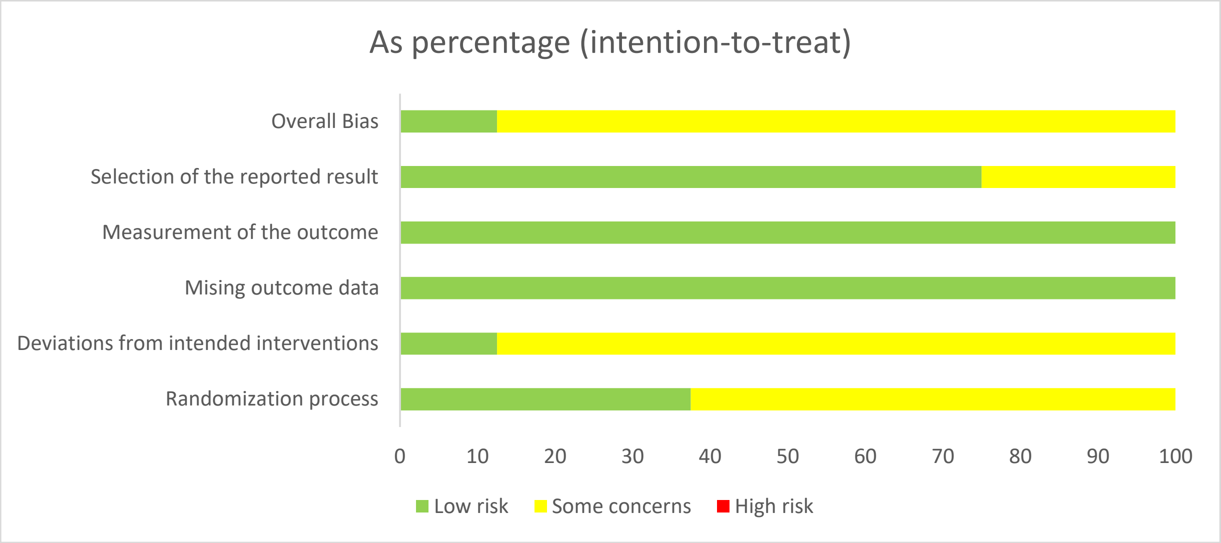

PFS

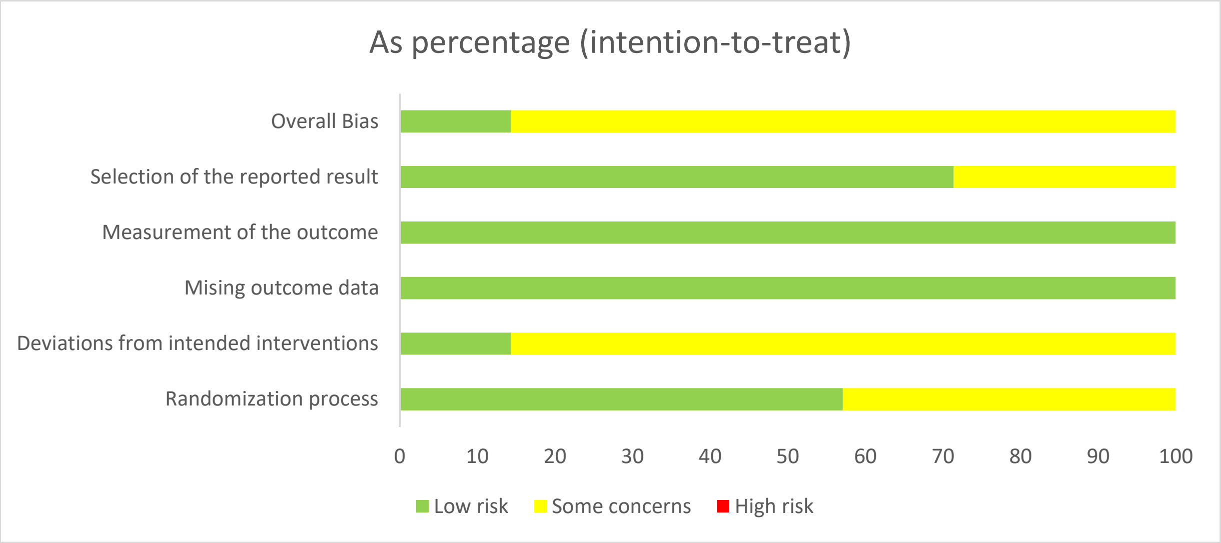

OS

b)

Intention-to-treat

Unique ID	Study ID	Experimental	Comparator	Outcome	Weight	D1	D2	D3	D4	D5	Overall
1	Wang et al. 2022	BR-Ibrutinib+R	BR+R	PFS, OS	1	+	+	+	+	+	+
2	Fischer et al. 2021	R-CHOP	CHOP	PFS, OS	1	!	!	+	+	!	!
3	Smith et al. 2021	BVR	BR	PFS	1	!	!	+	+	+	!
4	Flinn et al. 2019	BR	R-CHOP	PFS, OS	1	+	!	+	+	+	!
5	Robak et al. 2015	VR-CAP	R-CHOP	PFS, OS	1	+	!	+	+	+	!
6	Rule et al. 2016	R-FC	FC	PFS, OS	1	!	!	+	+	+	!
7	Rummel et al. 2016	BR+R	BR	PFS, OS	1	!	!	+	+	!	!
8	Rummel et al. 2013	BR	R-CHOP	PFS	1	!	!	+	+	+	!
9	Kluin-Nelemans et al. 2012	R-FC	R-CHOP	OS	1	+	!	+	+	+	!

+ Low risk
! Some concerns
\- High risk

D1 Randomisation process
D2 Deviations from the intended interventions
D3 Missing outcome data
D4 Measurement of the outcome
D5 Selection of the reported result

Supplementary Fig. S1 Results of risk of bias assessment

a)

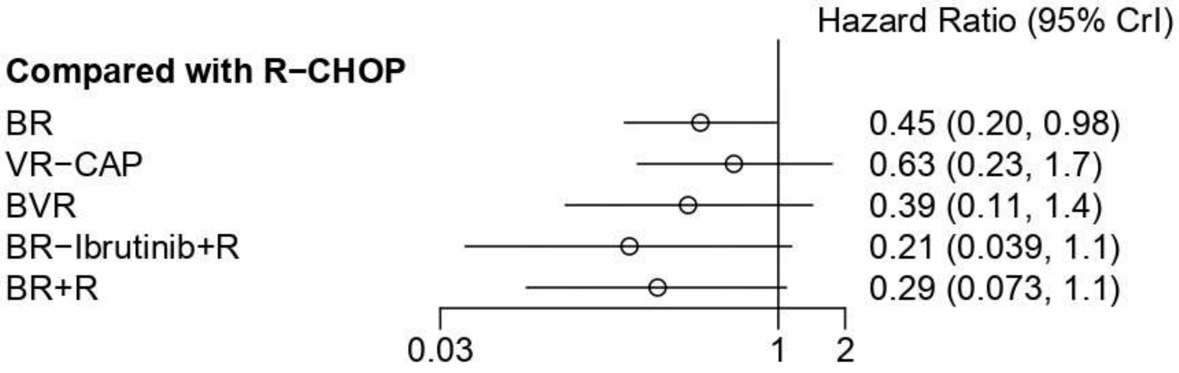

PFS

b)

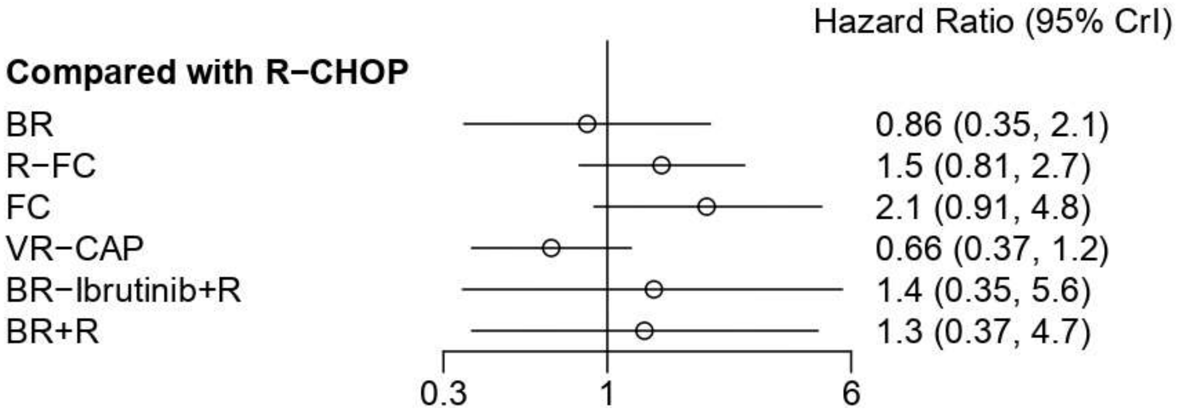

OS

Supplementary Fig. S2 Forest plot of sensitivity analysis (i)

a)

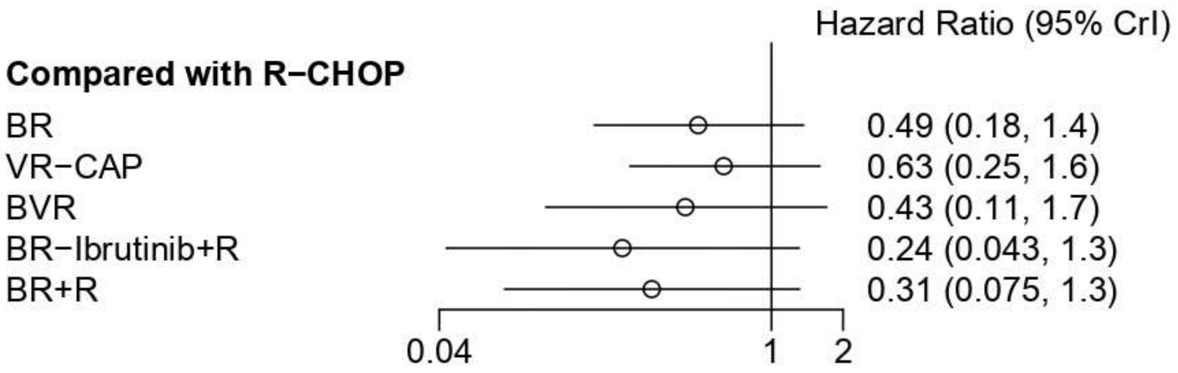

PFS

b)

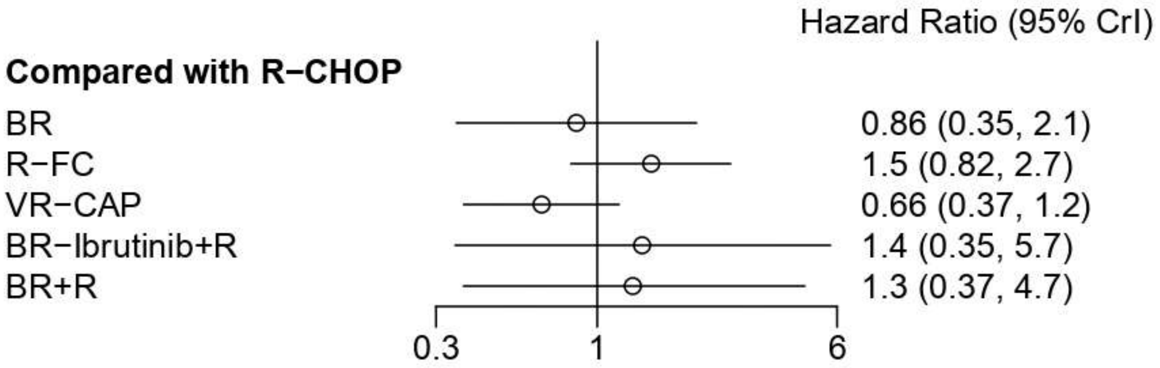

OS

Supplementary Fig. S3 Forest plot of sensitivity analysis (ii)

a)

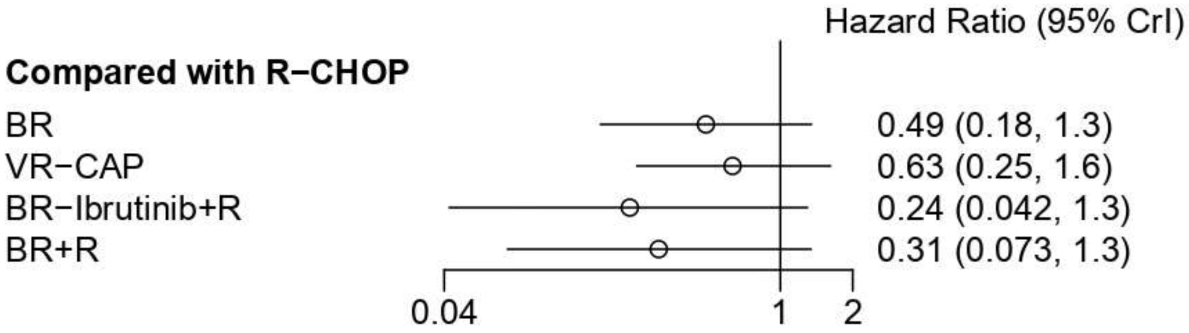

PFS

b)

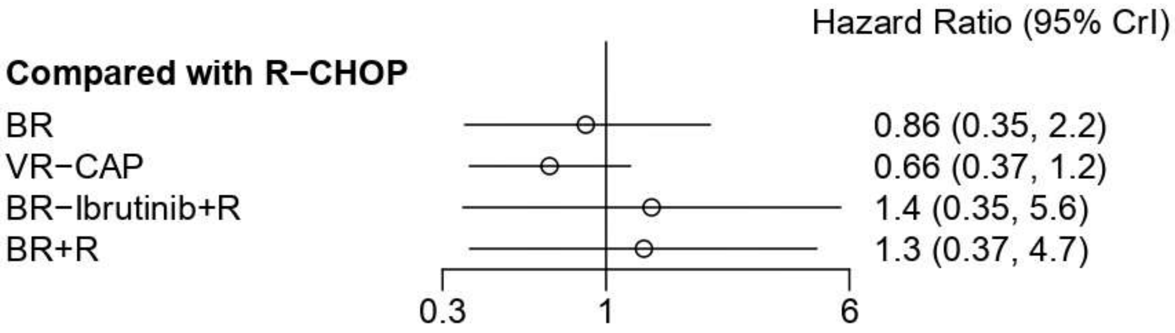

OS

Supplementary Fig. S4 Forest plot of sensitivity analysis (iii)
