# Supplementary material for: Efficacy of front‐line immunochemotherapy for transplant‐ineligible mantle cell lymphoma: A network meta‐analysis of randomized controlled trials
Source: Cancer Med. 2023 Jun 1;12(14):15107–16. doi: 10.1002/cam4.6183 (PMC10417079; doi:10.1002/cam4.6183)
Supplement: Supplementary file 2 — Table S1‐2. [file CAM4-12-15107-s002.docx]

| Regimen | SUCRA | | | PbBT | | |
| --- | --- | --- | --- | --- | --- | --- |
|  | (i) | (ii) | (iii) | (i) | (ii) | (iii) |
| BR-Ibrutinib+R | 0.88 | 0.87 | 0.88 | 70% | 68% | 73% |
| BR+R | 0.72 | 0.71 | 0.70 | 11% | 11% | 14% |
| BVR | 0.58 | 0.55 | NA | 13% | 12% | NA |
| BR | 0.47 | 0.45 | 0.47 | 2% | 2% | 5% |
| VR-CAP | 0.31 | 0.35 | 0.37 | 5% | 7% | 9% |
| R-CHOP | 0.05 | 0.07 | 0.07 | 0% | 0% | 0% |

SUCRA: surface under the cumulative ranking curve; PbBT, probability of being the best treatment.

(i) excluding the data from Fischer L et al. on the post hoc assessment of treatment effect between R-CHOP and CHOP;

(ii) from (i), further excluding the data from Flinn IW. et al. with small sample size; (iii) from (ii), further excluding the data from Smith MR. et al. because its HR and 95% CI were calculated by the method provided in the literature.

**Supplementary Table S1.** **Ranking of progression-free survival of immunochemotherapy regimens in sensitivity analyses**

| Regimen | SUCRA | | | PbBT | | |
| --- | --- | --- | --- | --- | --- | --- |
|  | (i) | (ii) | (iii) | (i) | (ii) | (iii) |
| VR-CAP | 0.88 | 0.86 | 0.83 | 63% | 63% | 64% |
| BR | 0.73 | 0.69 | 0.65 | 22% | 23% | 23% |
| R-CHOP | 0.61 | 0.54 | 0.45 | 2% | 2% | 2% |
| BR+R | 0.43 | 0.37 | 0.32 | 5% | 5% | 5% |
| BR-Ibrutinib+R | 0.37 | 0.31 | 0.26 | 6% | 6% | 6% |
| R-FC | 0.34 | 0.23 | NA | 1% | 1% | NA |
| FC | 0.13 | NA | NA | 1% | NA | NA |

SUCRA: surface under the cumulative ranking curve; PbBT, probability of being the best treatment.

(i) excluding the data from Fischer L et al.;

(ii) from (i), further excluding the data from Rule S et al. on the treatment effect estimation between R-FC and FC;

(iii) from (ii), further excluding the data from Kluin-Nelemans HC. et al. on the treatment effect estimation between R-FC and R-CHOP.

**Supplementary Table S2.** **Ranking of overall survival of immunochemotherapy regimens in sensitivity analyses**
